# Supplementary material for: Ehrlichia SLiM ligand mimetic activates Hedgehog signaling to engage a BCL-2 anti-apoptotic cellular program
Source: PLoS Pathog. 2022 May 16;18(5):e1010345. doi: 10.1371/journal.ppat.1010345 (PMC9135340; doi:10.1371/journal.ppat.1010345)
Supplement: Attachment — Submitted filename: Response to reviewer comments- PLoS.docx [file ppat.1010345.s001.docx]

April 8, 2022

Re: PPATHOGENS-D-22-00238 (*Ehrlichia* SLiM ligand mimetic activates Hedgehog signaling to

engage a BCL-2 anti-apoptotic cellular program)

Dear Editor,

Please find a revised version of manuscript PPATHOGENS-D-22-00238 “*Ehrlichia* SLiM ligand mimetic activates Hedgehog signaling to engage a BCL-2 anti-apoptotic cellular program”. We thank the reviewers for the insightful comments and suggestions, which have been very helpful in the revision of this manuscript. An item by item response to the comments is provided below.

**Responses to reviewers’ comments**

**Reviewer #1:**

**Reviewer Summary:** This study describes the identification and characterization of a Hedgehog pathway SLiM mimetic within the secreted Ehrlichia chaffeensis TRP120 effector. Authors identified the Hedgehog pathway SLiM mimetic through BLAST and ISM analysis. The Hedgehog pathway benefits E. chaffeensis replication and survival, as shown through iRNA of various pathway components. Through this knockdown analysis, PTCH2 was identified as being a potentially important target. Through fluorescence microscopy, TRP120 and PTCH2 were shown to colocalize in both THP-1 cells and HeLa cells. Direct protein-protein interactions between TRP120 and PTCH2 were demonstrated with reciprocal immunoprecipitations and SPR. E. chaffeensis infection was shown to induce the Hedgehog pathway through PCR arrays and assaying/imaging nuclear accumulation of the transcription factor GLI-1. Purified and recombinant TRP120 induces the Hedgehog pathway activation and nuclear accumulation of GLI-1 (THP-1 and primary human monocytes). The TRP120-Hh-SLiM was shown to be sufficient for inducing the Hedgehog pathway and blocking the peptide with specific antibodies abolished this induction. Finally, the TRP120-Hh-SLiM is shown to upregulate BCL-2 through the Hedgehog pathway, demonstrating a strategy that inhibits apoptosis and promotes the survival of E. chaffeensis.

This is an excellent and thorough investigation of a TRP120 SLiM mimetic that manipulates the Hedgehog pathway. Multiple approaches were used to confirm the mechanism of action, which is neatly tied back to E. chaffeensis’ virulence strategy and survival. Since this is the first reported eukaryotic Hedgehog pathway SLiM mimetic in a bacterial proteome, the findings are novel and significant for the field of intracellular pathogens. A few suggestions are noted to improve clarity of this manuscript.

**Response:** Thank you for your time and positive feedback. We have corrected errors in text and figures and explained text and figures that created confusion. Please see below.

**Comment 1:** *Figure 3A: it is unclear what the lower, left two frames are showing in the E. chaffeensis panel*

**Response:** We have removed these supplemental intensity distribution panels as they are not necessary for conclusions regarding colocalization that are based on the included PDM analysis.

**Comment 2:** *Figures 4A and 7A: For THP-1s, why does there appear to be an even greater concentration of GLI-1 around the outside perimeter of the nucleus when compared to nuclear accumulation levels (but not in primary human monocytes)?*

**Response:** GLI-1 accumulates in the cytoplasm and translocates to the nucleus in response to Hh signaling. Thus, the GLI-1 cytoplasmic and nuclear localization observed is consistent with canonical Hh activation. However, we agree that there is more nuclear accumulation of GLI-1 in primary monocytes compared to THP-1 cells, but the reason for this difference is not known. Nevertheless, nuclear GLI-1 levels in both cells are significantly higher in *E. chaffeensis-*infected, and TRP120 and TRP120 Hh SLiM-treated cells (see revised Figs. 4, 7 and 8).

**Comment 3:** *In general, there are many acronyms that make it somewhat difficult to follow the text. This reviewer suggests spelling out some acronyms to improve readability (e.g. “knockdown” vs. KD, “primary human monocytes” vs. PHM, “Hedgehog” vs Hh, etc.)*

**Response:** We have revised acronyms to improve readability.

**Comment 4:** *Line 160: What is dsb? Please spell out this acronym and/or explain.*

**Response:** We have defined *dsb* gene in the text.

**Comment 5:** *Line 248: typo. GL-1 should be GLI-1.*

**Response:** Corrected.

**Comment 6:** *Figure 8A: it looks like there may be some very faint GLI-1 signal from TRP120-TR (-), albeit significantly less than TRP120 or TRP120-Hh-SliM.*

**Response:** Although there appears to be a faint signal, quantitative analysis demonstrates no significant upregulation of TRP120-TR (-) compared to control cells.

**Comment 7:** *Line 292 and 316-317: consistent labeling throughout the manuscript is suggested: TRP120 Hh SLiM vs TRP120-Hh-SliM, BCL2 vs BCL-2, Hedgehog vs Hh.*

**Response:** We have corrected labeling to be consistent throughout.

**Comment 8:** *Line 340: please explain what JC-1 dye stains.*

**Response:** Further description of the JC-1 dye has been included in the methods section.

**Comment 9:** *Figure 12 E and F: bar graphs appear to be misaligned at the bottom and/or cut off*

**Response:** Sorry for the confusion. We have aligned the panels.

**Reviewer #2:**

**Reviewer Summary:** *The manuscript describes a study on Ehrlichia chaffeensis activation of Hedgehog signaling via TRP120. The authors show that TRP120 has sequence and functional similarity to Hedgehog ligands and identify a candidate ligand SLiM. Confirmation of E. chaffeensis activation of Hedgehog signaling is done by siRNA depletion, Co-IP and plasma resonance. Results show that depletion of Hedgehog signaling negatively affects E. chaffeensis reproduction. rTRP120-TR is shown to directly interact with Patched 2. Infection by E. chaffeensis upregulates Hedgehog signaling and target genes. Additional experiments confirm the interaction of SLIM in nuclear locations of GLI-1 and expansion of Hedgehog signaling. Inhibition of Hedgehog signaling increased pro-apoptosis in infected cells.*

*Overall, the manuscript is thorough, well-written, includes controls and rigor throughout. The mechanisms described are highly novel and provide significant insights into E. chaffeensis infection strategies. Mechanisms of immune evasion by intracellular pathogens such as E. chaffeensis are key to understanding how infections progress and are maintained in the host. As such, the description of how Hedgehog signaling is activated during infection and contributes to inhibition of apoptosis is highly novel and significant within the field.*

**Response:** Thank you for the positive feedback and for highlighting the significance of these findings. We have corrected errors in text and figures and explained text and figures that created confusion. Please see below.

**Comment 1:** *The authors show that GLI-1 activation is increased during infection. However, it is not addressed as to why all cells appear to have GLI-1 activation, even cells that do not appear to be infected by E. chaffeensis. This question is based on data presented in Figure 4.*

**Response:** We infected with *E. chaffeensis* at an MOI of 100 to obtain infection of all cells. Enlarging images with zoom tool enables one to visualize that all cells indeed contain ehrlichiae as determined by anti-Dsb *Ehrlichia* antibody (red). We have also increased the TRITC signal to enhance Dsb staining to make it more visible. Furthermore, the images were obtained by confocal microscopy. Therefore, these images represent a small slice of the cell and only ehrlichiae that are present in a given section may be visualized and not all ehrlichiae in a given cell.

**Comment 2:** *Line 128: Define ISM here instead of line 139*

**Response:** We have defined ISM in the text.

**Comment 3:** *Line 160: explain what dsb encodes for and how this correlates to infection status*

**Response:** We have defined Dsb and provided further explanation in the text.

**Comment 4:** *Figure 1: writing within the figure is hard to see. Increase font of "NTD, PR and CTD". NTD and CTD not defined in figure legend. I assume it is N-terminal and C-terminal? Please fix for consistency.*

**Response:** The figure legend and figure have been corrected. We also increased the font size and description to make it easier to read.

**Comment 5:** *Figure 3, 7, 8, 9, 11: please add scale bar to microscopy images and figure legends*

**Response:** We apologize for the confusion. All figures and legends show scale bar, but we placed the scale bar in only the first image of each grouping at the bottom left corner to avoid redundancy.

**Comment 6:** *Figure 4B: The representative image in 4B barely shows on small "dot" corresponding to dsb. This is quite different than the above panel at 10 hours.*

**Response:** There are fewer primary human monocytes compared to THP-1 cells in 4A; however, in Fig 4B, all cells contain ehrlichiae as determined by Dsb staining. The Dsb is easier to visualize on a computer monitor.

**Comment 7:** *Figure 7: Scale bar is mentioned in the figure legends but missing from figure.*

**Response:** We apologize for the confusion. Fig. 7 shows a scale bar, but we placed the scale bar in the first image of each grouping at the bottom left corner.

**Comment 8**: *Figure 11: It is not clear what MOI was used in these experiments? Based on the images provided in the microscopy insets it appears that there are multiple morulae in each cell. The data provided just states the number of cells that were healthy vs apoptic but how many of those cells in each population were infected? It is also unclear as to how many cells were counted in each condition.*

**Response:** An MOI of 50 or 100 was used in experiments and is described in the individual figure legends and methods. In Fig. 11, we infected with *E. chaffeensis* at an MOI of 100 to obtain infection of all cells. Additionally, we provided further explanation on how cells were counted in the figure legend.

**Comment 9***. Figure 13: this is a very nice model of how signaling is being altered during infection; however, the key player: E. chaffeensis, is missing in the figure and needs to be added in. How do the authors predict that the bacteria fall in this mechanism?*

**Response:** We appreciate your suggestion and have added *E. chaffeensis* to the figure.

**Reviewer #3:**

**Reviewer Summary:** *Reviewer #3: This is a very interesting study that further defines TRP120 activity during Ehrlichia infection. This versatile protein is shown to trigger hedgehog signaling that impacts infection, gene expression, and apoptosis. The manuscript is logically presented and shows data that will be important for the Ehrlichia field, possibly extending to the intracellular pathogen field at large. I have several comments that, while mostly minor, should strengthen the authors' conclusions.*

**Response:** Thank you for your time and positive feedback. We have corrected errors in text and figures and explained text and figures that created confusion. Please see below.

**Comment 1:** *Fluorescence microscopy results should be quantified throughout to enhance the ability to make clear conclusions.*

**Response:** We appreciate the suggestion. We have added quantification for confocal microscopy.

**Comment 2:** *Do not use the words "strong" and "weak" when referring to protein-protein interactions. Refer to affinities instead.*

**Response:** We appreciate your suggestion and have revised the text accordingly.

**Comment 3:** *Be careful when saying that a protein is activated based on localization in a cell. A downstream readout is critical (and often provided later in the manuscript) to make this claim.*

**Response:** We appreciate your suggestion and described GLI-1 as being upregulated and the Hh pathway as being activated based on downstream readout of gene transcription.

**Comment 4:** *Lines 146-147 - cannot make claims about sharing biological functions based solely on in silico analyses.*

**Response:** We have revised the text.

**Comment 5:** *Lines 171-172 - cannot make claims about interactions based on siRNA knockdown.*

**Response:** We have revised the text.

**Comment 6:** *Lines 250-252 - I disagree with this conclusion based on the data shown in Fig. 7. Clear activation is not apparent.*

**Response:** We have included intensity graphs of the nucleus demonstrating a significant increase of GLI-1 in the nucleus compared to the control.

**Comment 7:** *Lines 524-526 - this study did not directly test whether TRP120 prevents apoptosis via Bcl-2. This statement is premature.*

**Response:** TRP120 has been shown to directly activate Hh signaling and an inhibitor of Hh signaling prevents apoptosis. Thus, we believe that one can conclude that TRP120 through Hh activation is responsible for inhibiting apoptosis through BCL-2. However, we have revised the text to address this concern.

**Comment 8:** *Figure 3 - PDM should be defined. Colors in panel D should be defined. In panel C, a non-interacting protein control should be included to ensure that PTCH2 is not just "sticky" and binding non-specifically to TRP120.*

**Response:** The figure legend and figure have been revised to define PDM and colors in panel D. An additional control has also been included.

**Comment 9:** *Figure 4 - THP-1 images are unclear as presented. It appears that GLI-1 is actually absent in the nucleus of many cells.*

**Response:** We have included intensity graphs of the nucleus demonstrating a significant increase of GLI-1 in the nucleus compared to negative control.

**Comment 10:** *Figure 6 - SUFU blot results disagree with Fig. 5B results. This should be explained. In panel A, N and C need to be defined.*

**Response:** It is generally known that gene transcription and protein levels do not always correlate for many reasons. Thus, it is not unexpected that *SUFU* gene transcription and protein levels may not agree. One potential explanation for this discrepancy is that *E. chaffeensis* is known to interact with and degrade host proteins during infection which could be occurring with SUFU. Hence, the increased *SUFU* gene expression observed at 48 h could be compensatory for the decreased levels of SUFU protein we detected. We have defined N and C in panel A.

**Comment 11:** *Figure 7 - Panel A - again the THP-1 results are unclear. GLI-1 appears to be present mainly in the cytoplasm. For comparison, Figure 9 microscopy is much clearer and more convincing regarding nuclear localization of GLI-1. Nuclear fractionation and immunoblots would strengthen claims.*

**Response:** We have included intensity graphs of the nucleus demonstrating a significant increase of GLI-1 in the nucleus compared to control. We also demonstrate that rTRP120 regulates GLI-1 similar to the endogenous ligand rShh (positive control).

**Comment 12:** *Figure 11 - to fully accept that model, TRP120 effects on apoptosis in the presence/absence of Vismodegib must be shown.*

**Response:** As mentioned above, TRP120 Hh SLiM has been shown to directly activate Hh signaling and an inhibitor of Hh signaling prevents apoptosis. Thus, we believe that one can conclude that TRP120 through Hh activation is responsible for inhibiting apoptosis through BCL-2 (Fig. 10, TRP120 Hh SLiM upregulates BCL2). However, we have revised the text eliminate direct reference of TRP120 to apoptosis.
